# Transient Receptor Potential Canonical 5-Scramblase Signaling Complex Mediates Neuronal Phosphatidylserine Externalization and Apoptosis

**DOI:** 10.3390/cells9030547

**Published:** 2020-02-26

**Authors:** Jizheng Guo, Jie Li, Lin Xia, Yang Wang, Jinhang Zhu, Juan Du, Yungang Lu, Guodong Liu, Xiaoqiang Yao, Bing Shen

**Affiliations:** 1 School of Basic Medical Sciences, Anhui Medical University, Hefei, Anhui 230032, China; guojizheng@ahmu.edu.cn (J.G.); lijiedancy@163.com (J.L.); rogerxl@163.com (L.X.); brucew_123@163.com (Y.W.); zhujinhang@ahmu.edu.cn (J.Z.); dujuan@ahmu.edu.cn (J.D.); 2 Brown Foundation Institute of Molecular Medicine, University of Texas, McGovern Medical School, Houston, TX 77030, USA; yunganglu@uth.tmc.edu; 3 School of Biomedical Sciences, Chinese University of Hong Kong, Hong Kong, China; 4 Institute of Biomedical and Health, School of Life and Health Science, Anhui Science & Technology University, Fengyang, Anhui 233100, China; guodong.liu@ndsu.edu

**Keywords:** apoptosis, cerebral ischemia reperfusion, PLSCR1, phosphatidylserine, scramblase, TRPC5

## Abstract

Phospholipid scramblase 1 (PLSCR1), a lipid-binding and Ca^2+^-sensitive protein located on plasma membranes, is critically involved in phosphatidylserine (PS) externalization, an important process in cell apoptosis. Transient receptor potential canonical 5 (TRPC5), is a nonselective Ca^2+^ channel in neurons that interacts with many downstream molecules, participating in diverse physiological functions including temperature or mechanical sensation. The interaction between TRPC5 and PLSCR1 has never been reported. Here, we showed that PLSCR1 interacts with TRPC5 through their C-termini in HEK293 cells and mouse cortical neurons. Formation of TRPC5-PLSCR1 complex stimulates PS externalization and promotes cell apoptosis in HEK293 cells and mouse cerebral neurons. Furthermore, in vivo studies showed that PS externalization in cortical neurons induced by artificial cerebral ischemia-reperfusion was reduced in TRPC5 knockout mice compared to wild-type mice, and that the percentage of apoptotic neurons was also lower in TRPC5 knockout mice than in wild-type mice. Collectively, the present study suggested that TRPC5-PLSCR1 is a signaling complex mediating PS externalization and apoptosis in neurons and that TRPC5 plays a pathological role in cerebral-ischemia reperfusion injury.

## 1. Introduction

Phosphatidylserine (PS), along with phosphatidylethanolamine and phosphatidylinositol, is located mainly in the inner leaflet, whereas sphingomyelin and phosphatidylcholine are located primarily in the outer leaflet of the cell membrane [1]. Phospholipid scramblases are Ca^2+^-dependent but ATP-independent enzymes that play a key role in the collapse of this asymmetrical distribution of phospholipids between the two leaflet of cell membrane [1,2]. This collapse of asymmetrical distribution of phospholipids between the inner and outer leaflets of the cell membrane plays important roles in many physiological processes, for example, in blood coagulation and cell apoptosis. Phospholipid scramblase (PLSCR) family of gene, including PLSCR1-5, is among the main molecular candidates of phospholipid scramblases [3]. Although the lipid scrambling activity of PLSCR family remains under debate, numerous reports have demonstrated that PLSCR1 can induce PS externalization in a Ca^2+^-dependent manner both in vivo and in vitro [3,4,5], and is critically involved in cell apoptosis [6].

PLSCR1 contains a nuclear localization signal, a DNA-binding domain, a proline-rich N-terminal region, and a conserved Ca^2+^-binding domain [7,8,9]. Hence, PLSCR1 is sensitive to regional Ca^2+^ changes. A previous study reported that PLSCR2 fused with the proline-rich domain of PLSCR1 can acquire responsiveness to Ca^2+^ [10]. The expression of PLSCR1 is modulated by interferon, epidermal growth factor, leukemic cell differentiation-inducing agents, and SNAIL, a transcriptional regulator of epithelial–mesenchymal transition [11,12]. Dysregulation of PLSCR1 may contribute to many human diseases. For example, elevated expression of PLSCR1 is implicated in systemic lupus erythematosus and antiphospholipid syndrome [13,14], and increasing PLSCR1 mRNA expression is also suggested to increase the survival rate in acute promyelocytic leukemia and acute myelogenous leukemia patients [15,16]. Reports also showed that PLSCR1 is an oncogenic molecule in ovarian, colorectal, and metastatic liver cancers [17,18,19,20], and that PS externalization plays a role in eryptosis and blood coagulation in red blood cells [21,22].

Transient receptor potential canonical 5 (TRPC5), one of the TRP family proteins, is a nonselective Ca^2+^-permeable channel highly expressed in the central nervous system, with moderate or low expression in liver, heart, vasculature and kidney [23,24]. TRPC5 has six helical transmembrane segments with intracellular amino and carboxyl terminals. There are diverse modes of activation for TRPC5 channels. The channels can be activated by G-protein-coupled receptors, lanthanides, lysophosphatidylcholine, cold and membrane stretch [25,26,27,28,29,30]. Functionally, the channel plays important roles in a variety of body functions including neuronal growth cone extension [31], animal fear behavior [32], cold detection [30], cancer cell chemoresistance [33,34], baroreceptor pressure-sensing and endothelium-dependent vascular contraction [35,36]. Interestingly, two studies indicated that TRPC5-mediated Ca^2+^ signaling contributes to neuronal cell apoptosis [37,38]. However, the underlying molecular mechanism for TRPC5 involvement in cell apoptosis remains obscure.

In the present study, we hypothesized that PLSCR1 associates with TRPC5 to form a functional signaling complex, contributing to Ca^2+^-dependent phosphatidylserine externalization and apoptosis in neurons. Multiple techniques, including fluorescence resonance energy transfer (FRET), in situ proximity ligation (PLA), and co-immunoprecipitation assays, were used to determine whether TRPC5 physically interacts with PLSCR1. TRPC5 knockout (TRPC5 KO) mice were used to explore the possible functional roles of TRPC5 and PLSCR1 in PS externalization and cerebral neuron apoptosis.

## 2. Materials and Methods

### 2.1. Materials and Solutions

Daidzein, LaCl_3,_ and In situ PLA kits were purchased from Sigma-Aldrich (St. Louis, MO, USA). Mouse anti-DsRed2 (sc-101526), rabbit anti-GFP (sc-5385), and goat anti-PLSCR1 (sc-27782) antibodies were obtained from Santa Cruz Biotechnology (Dallas, TX, USA). Rabbit anti-TRPC5 (ACC-020), anti-TRPC4 (ACC-018) antibodies were purchased from Alomone Labs (Jerusalem, Israel). Rabbit anti-TRPC1 antibody was purchased from Bioss Company (bs-10404R, Beijing, China). The annexin V-FITC apoptosis detection kit was obtained from BestBio Company (BB-4101, Nanjing, China). The TUNEL BrightRed apoptosis detection kit was purchased from Vazyme Company (A113-01, Nanjing, China). The mouse TRPC5 cDNA (NM_009428.2) was subcloned into either pmCherry-N1 or pmCherry-C1 vectors. Human PLSCR1 cDNA (NM_021105.1) was subcloned into either pEGFP-N1 or pmCherry vectors. The dominant-negative TRPC5 construct (DN-TRPC5) was a gift from D Clapham from Harvard University and subcloned into the pmCherry-N1 vector [39]. The EGFP-mCherry plasmid was prepared by inserting mCherry into pEGFP-C1 at the *Xho*I site. NPSS (pH 7.4) contained (in mmol/L) 140 NaCl, 5 KCl, 2 MgCl_2_, 1 CaCl_2_, 10 glucose, and 10 HEPES. The hypotonic bath solution (pH 7.4) contained (in mmol/L) 65 NaCl, 5 KCl, 1 CaCl_2_, 1 MgCl_2_, and 10 HEPES; osmolarity was adjusted to 210 mOsm. The artificial cerebrospinal fluid (ACSF, pH 7.4) contained (in mmol/L) 118 NaCl, 2.5 KCl, 3 MgSO_4_, 1.1 NaH_2_PO_4_, 26 NaHCO_3_, 1 CaCl_2_, and 11 D-glucose.

### 2.2. Cell Culture and Transfection

The human embryonic kidney 293 (HEK293) cells were purchased from the American Type Culture Collection (Manassas, VA, USA) and cultured in Dulbecco’s modified Eagle’s medium (DMEM) at 37 °C in an incubator with 5% CO_2_. Plasmid transfections were performed by Lipofectamine 2000 as previously described [40]. TRPC5 (including mCherry-TRPC5 and TRPC5-mCherry) and PLSCR1 (including PLSCR1-EGFP, PLSCR1-mCherry and PLSCR1) plasmids were used in the study. The HEK293 cells were cultured in six-well plates with 500 μL culture medium without fetal bovine serum (FBS) and antibiotics in each well. The cells were grown to approximately 80% confluency. The plasmid was diluted in 50 μL of Opti-MEM reduced-serum medium. Lipofectamine 2000 (2 μL) was also diluted in 50 μL of Opti-MEM medium before use. After a 5-min incubation, the two diluted liquids were combined, mixed, and incubated together for 20 min at room temperature. Finally, the plasmid–Lipofectamine 2000 complexes were added to the HEK293 cells. The medium was changed to DMEM supplemented with FBS after 6 h.

### 2.3. Detection of PS Externalization using Annexin V-FITC Assay

Annexin V is a Ca^2+^-dependent phospholipid-binding protein that has a high affinity for PS. It is widely used to identify cells undergoing apoptosis with exposed PS. FITC, a fluorochrome conjugated to annexin V, serves as a sensitive probe for flow cytometric analysis. An annexin V-FITC apoptosis detection kit was used to detect PS externalization. Briefly, HEK293 cells were pretreated with NPSS (control), hypotonic solution, daidzein (100 μmol/L), or LaCl_3_ (100 μmol/L) for 15 min at 37 °C in an incubator. The cells were then washed twice with cold NPSS and incubated with 300 μL of binding buffer (0.01 M HEPES/NaOH at pH 7.4, 0.14 M NaCl, 2.5 mM CaCl_2_). Annexin V-FITC (5 μL) was added to the cells and gently mixed and incubated for 15 min at room temperature in the dark. Finally, another 200 μL of binding buffer was added to the cells. The resultant images were visualized using a FluoView FV1000 confocal microscope system (Olympus, Tokyo, Japan).

### 2.4. Mouse Model of Cerebral Ischemia-Reperfusion

All animal experiments were performed according to NIH publication no. 8523 and were approved by the Animal Experimentation Ethics Committee of the Chinese University of Hong Kong. Longa et al. established a regional cerebral ischemia model [41]. We modified their method to successfully establish a mouse model of cerebral ischemia. The normal C57, wild-type and TRPC5 KO mice were intraperitoneally injected with 5% (*w*/*v*) chloral hydrate (300 mg/kg) for anesthesia and fixed in the supine position [42]. The neck of the mouse was incised at the midline to dissect the right common carotid artery, external carotid artery, and internal carotid artery (ICA). An microvascular clip was used to temporarily block the ICA, a small cut was made at the common carotid artery from the bifurcation 3–4 mm, and a thread embolus (2.0 cm nylon suture with diameter 0.15 mm and its tip rounded by heating) was inserted to the artery through the external carotid stump and advanced to a point about 10 mm to the carotid bifurcation. After the ICA clip was released, the thread embolus was moved forward and fixed using a gel at the start of the middle cerebral artery (MCA) to occlude MCA blood flow. After 60 min of occlusion, the thread embolus was carefully removed to restore reperfusion for 24 h. Sham control mice were performed by identical surgery, but did not have the thread embolus inserted. During this surgery, the body temperature of the mouse was controlled between 25 °C to 28 °C and the vagus nerve was protected to avoid additional influence on MCA blood flow.

### 2.5. Preparation of Mouse Brain Sections

The normal C57, wild-type and TRPC5 KO mice were killed by cervical dislocation. Whole mouse brains were isolated and preserved in ACSF oxygenated with 95% O_2_ mixed with 5% CO_2_. The brains were coronally sliced into 300-μm-thick sections. The sections were cultured in six-well plates with 1 mL of ACSF for 20 min to balance the tissues in new environment and then treated with ACSF (control), hypotonic solution, daidzein (100 μmol/L) or LaCl_3_ (100 μmol/L) for 15 min at 37 °C in an incubator with 5% CO_2_. Annexin V–FITC (5 μL) was added to each well and the sections were incubated for an additional 10 min. Finally, each well was washed with ACSF three times and cultured under the respective solutions again for next image capture. The images were captured using a FluoView FV1000 confocal microscope system within 30 min after the treatments. T5E3 (15 µg/mL), to block TRPC5 channels, and IgG (15 µg/mL), used as a control, were added to the brain sections obtained from normal C57 mice for 30 min before the test solutions were added.

### 2.6. TUNEL Assay

The TUNEL assay to detect DNA fragmentation was performed according to the manufacturer’s instructions to determine the percentage of apoptotic cortical neurons. This assay is based on the nicks in the DNA specifically identified by TdT. This enzyme catalyzes the addition of dUTPs labeled with a fluorescent marker. The normal C57, wild-type and TRPC5 KO mice cerebral slices were fixed in a 4% paraformaldehyde solution for 25 min at 25 °C. After being washed three times, the neurons in the slices were permeabilized by a solution of 0.1% Triton X-100 and 0.1% sodium citrate for 5 min at room temperature. After equilibrating for 30 min in equilibration buffer at room temperature, the slices were treated with BrightRed Labeling Mix (TdT and 12-dUTP) for 1 h at 37 °C in a humidified and dark chamber. After the reaction, neuronal nuclei were stained with 4’,6-diamidino-2-phenylindole (DAPI). Finally, apoptotic (red; at a wavelength of 620 nm) and total neurons (blue; at a wavelength of 460 nm) cells were counted using a FluoView FV1000 confocal microscope system (Olympus, Tokyo, Japan). The percentage of apoptotic neurons was calculated as follows: (apoptotic cell number/total cell number) × 100.

### 2.7. Co-Immunoprecipitation and Immunoblots

Co-immunoprecipitation and immunoblot assays were performed as previously described [43]. Briefly, total proteins were extracted in a buffer at pH 8.0 containing 1% (*v*/*v*) Nonidet P-40, 150 mmol/L NaCl, 20 mmol/L Tris-HCl, and protease inhibitor cocktail tablets. A total of 800 µg of the extracted protein was mixed with 3 µg of anti-mCherry or anti-EGFP (HEK293 cells), or anti-PLSCR1 or anti-TRPC5 (mouse cerebral neurons) antibodies on a rocker overnight at 4 °C. On the next day, protein A magnetic beads were added and incubated for 3 h at 4 °C. The products of the co-immunoprecipitation were washed with protein extract buffer three times. Sodium dodecyl sulfate polyacrylamide gel electrophoresis (8% gels) was used to resolve the immunoprecipitates. The proteins were transferred to polyvinylidene difluoride membranes and incubated with primary antibodies at 1:250 dilution and 4 °C overnight in PBST buffer (PBS mixed with 0.1% Tween 20 and 5% nonfat dry milk). Finally, the membrane was incubated with a horseradish peroxidase-conjugated secondary antibody. An ECL system was used to visualize the target proteins.

### 2.8. Förster Resonance Energy Transfer (FRET) Assay

Sensitized emission FRET was performed according to the Leica confocal software manual. For the EGFP-mCherry FRET pair, EGFP was the donor fluorophore and mCherry was the acceptor fluorophore to be stimulated by EGFP emission [44]. PLSCR1 and TRPC5 were tagged with EGFP and mCherry, respectively. In the control group, cells with the donor only were transfected with the GFP-tagged construct and cells with the acceptor only were transfected with the mCherry-tagged as references. The references were used to obtain calibration coefficients and correct for excitation and emission cross talk. The FRET efficiency was recorded in HEK293 cells co-transfected with EGFP-tagged and mCherry-tagged constructs and calculated using the following equation [45]: E_FRET_ = (B-A×β-C×γ)/C, where A, B, and C are donor, FRET, and acceptor channel intensities, respectively. For the calibration factors, β was the FRET channel intensity/donor channel intensity in the cells with the donor only, and γ was the FRET channel intensity/acceptor channel intensity in the cells with the acceptor only.

### 2.9. PLA

The detection of the interaction between PLSCR1 and TRPC5 in native neurons by using a PLA kit was performed as previously described [43]. Freshly isolated normal C57 mouse cerebral cortical neurons were seeded on coverslips, fixed, permeabilized, and blocked with the Duolink blocking solution. Subsequently, neurons were incubated in the Duolink antibody diluent with goat anti-PLSCR1 and rabbit anti-TRPC5 antibodies (1:40) overnight at 4 °C. The control group was incubated under the same conditions but with only the rabbit anti-TRPC5 antibody. Following washes with physiological saline, neurons were incubated with the Duolink secondary antibodies conjugated with oligonucleotides (anti-goat PLA probe Plus, Cat. no. DUO92003; anti-rabbit PLA probe Minus, Cat. no. DUO92005) in a preheated, humidified chamber for 1 h at 37 °C. Then, neurons were incubated with a ligation solution consisting of two complementary oligonucleotides and one ligase. The proximity-dependent hybridization was followed by a rolling-circle amplification reaction, producing a repeated sequence product to amplify the signal. Finally, a fluorescence (Texas Red)-labeled complementary oligonucleotide detection probe was used to resolve the amplification products. The Duolink mounting medium containing DAPI as a nuclear stain was used to mount the neurons. The results were visualized using a FluoView FV1000 confocal microscope system.

### 2.10. Statistical Analysis

Statistical analyses were performed with a two-tailed unpaired Student’s *t* test or two-way ANOVA followed by the Tukey’s post-hoc comparison test when more than two treatments were compared. All data shown represent the results obtained from three independent experiments with standard errors of the mean (mean ± s.e.m). *p*-values < 0.05 were considered statistically significant.

## 3. Results

### 3.1. Spatial Proximity and Association of TRPC5 and PLSCR1 on HEK293 Plasma Membrane

**Figure 1:**
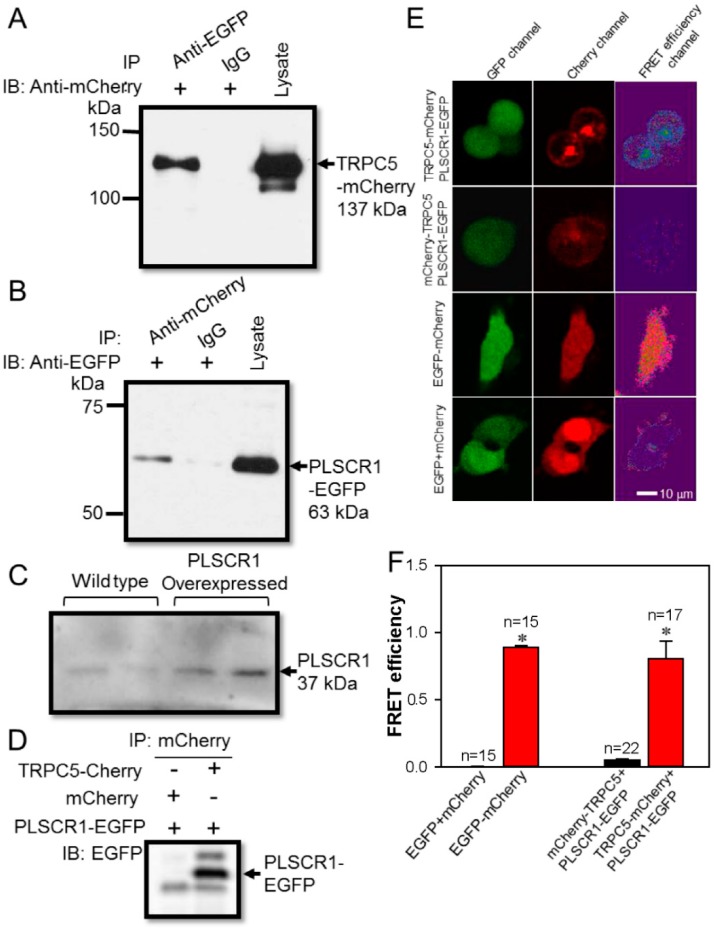
Association between TRPC5 and PLSCR1 in HEK293 cells. (**A**, **B**) Representative images of co-immunoprecipitation experiments in TRPC5-mCherry and PLSCR1-EGFP co-expressing HEK293 cells. (**A**): IP, anti-EGFP antibody or IgG; IB, anti-mCherry antibody. (**B**), IP, anti-mCherry antibody or IgG; IB, anti-EGFP antibody. Immunoblots of cell lysates were also shown on the right. (**C**) Representative images showing the expression of PLSCR1 in wild-type and PLSCR1-overexpressing HEK293 cells. (**D**) Representative images of co-immunoprecipitation experiments in HEK293 cells co-expressed with mCherry plus PLSCR1-EGFP, or with TRPC5-mCherry plus PLSCR1-EGFP. IP, mCherry antibody; IB, anti-EGFP antibody. (**E**) Representative images showing fluorescence signals in a FRET assay. HEK293 cells were overexpressed with different constructs as indicated. TRPC5-mCherry, mCherry tagged at the carboxyl terminus of TRPC5; mCherry-TRPC5, mCherry tagged at the amino terminus of TRPC5; PLSCR1-EGFP, EGFP tagged at the carboxyl terminus of PLSCR1. The EGFP emission signal was detected as green fluorescence (GFP channel), while the mCherry emission signal was detected as red fluorescence (Cherry channel). The FRET efficiency fluorescence was shown in the FRET efficiency channel. (**F**) Summary of data showing the differences in FRET efficiency. Values are shown as the mean ± SEM (n = 15–22); *p < 0.05 for EGFP + mCherry vs. EGFP-mCherry, or mCherry-TRPC5 + PLSCR1-EGFP vs. TRPC5-mCherry + PLSCR1-EGFP with a two-tailed unpaired Student’s *t* test.

To identify protein-protein interaction we used co-immunoprecipitation experiment. Here, we co-transfected TRPC5-mCherry and PLSCR1-EGFP plasmids into HEK293 cells. We used anti-EGFP to immuno-precipitate PLSCR1-EGFP. It pulled down TRPC5, as indicated by immunoblot with anti-mCherry antibody (Figure 1A). In reverse pull-down experiments, we used anti-mCherry to immune-precipitate TRPC5-mCherry. It pulled down PLSCR1, as indicated by immunoblot with anti-EGFP antibody (Figure 1B). In the control group, the primary antibody was replaced by preimmune IgG, and as expected, preimmune IgG failed to pull down either TRPC5-mCherry or PLSCR1-EGFP (Figure 1A,B). The specificity of the anti-PLSCR1 antibody was verified in PLSCR1-overexpressing HEK293 cells. The result indicated that PLSCR1 overexpression by transfected with PLSCR1 plasmid obviously increased the expression of PLSCR1 (Figure 1C). As another control, HEK293 cells were co-transfected with mCherry and PLSCR1-EGFP. As expected, co-immunoprecipitation with anti-mCherry antibody failed to pull down PLSCR1-EGFP, suggesting that there was no non-specific interaction between mCherry and PLSCR1-EGFP (Figure 1D).

To further elucidate the interaction of TRPC5 and PLSCR1, we conducted a FRET assay to identify protein-protein spatial proximity, capable of detecting the very close distance between EGFP and mCherry proteins of less than 10 nm [44,46]. PLSCR1 has an intracellular carboxyl terminal, whereas both the carboxyl and amino terminals of TRPC5 are located intracellularly. Thus, we attempted to determine whether the carboxyl terminus or the amino terminus of TRPC5 could be in close proximity to PLSCR1. Here, mCherry was tagged either to the carboxyl terminus of TRPC5 (TRPC5-mCherry) or tagged to the amino terminus of TRPC5 (mCherry-TRPC5), EGFP was tagged to the carboxyl terminus of PLSCR1 (PLSCR1-EGFP). We observed high FRET efficiency in HEK293 cells co-transfected with TRPC5-mCherry and PLSCR1-EGFP, but not in cells co-transfected with mCherry-TRPC5 and PLSCR1-EGFP (Figure 1E–F). 

In a positive control in which the cells were transfected with EGFP-mCherry concatemer, high FRET efficiency was detected. In a negative control, in which the cells were co-transfected with EGFP and mCherry as separate construct, no FRET signal was observed (Figure 1E–F). Taken together, these results indicated that the carboxyl but not the amino terminal of TRPC5 is closely associated with the carboxyl terminal of PLSCR1.

### 3.2. TRPC5 Promotes PS Externalization in HEK293 Cells

PS externalization was visualized using annexin V-FITC as a green fluorescence signal, while TRPC5 and PLSCR1 were visualized as red fluorescence signals because of the mCherry protein in their carboxyl terminals. Previous study from Schaefer et al. first indicated that LaCl_3_ is capable of activating TRPC5 [27].

**Figure 2:**
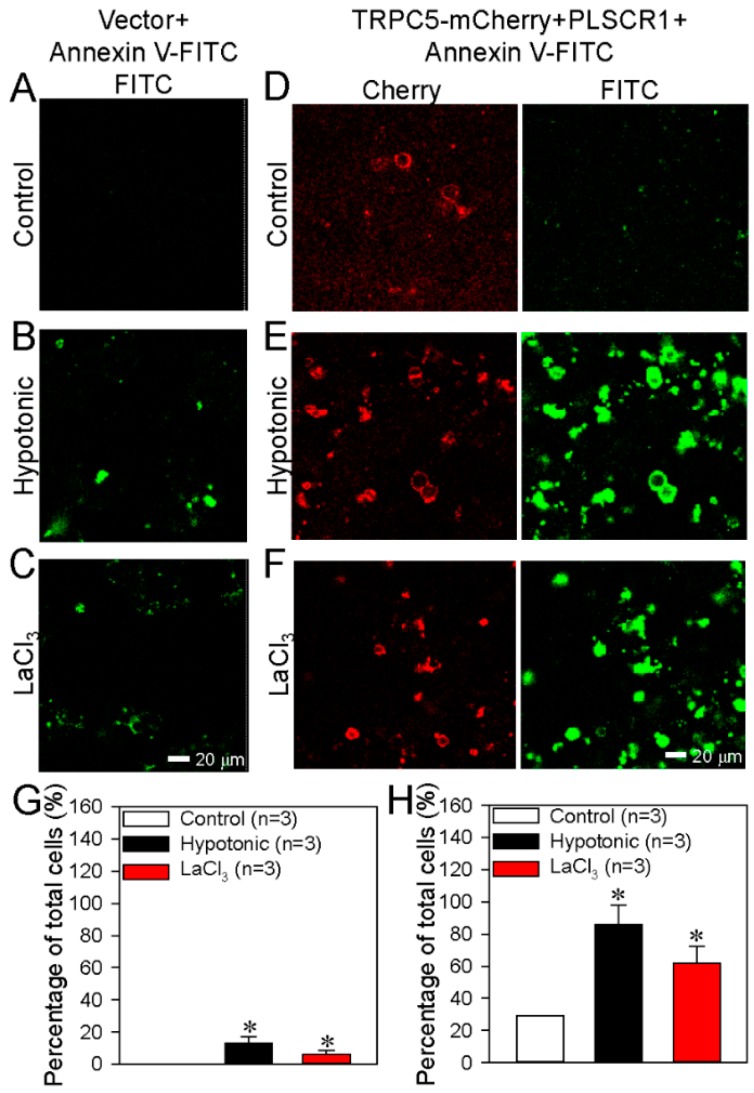
TRPC5+PLSCR1 stimulates phosphatidylserine (PS) externalization in HEK293 cells. (**A**–**F**) Representative images showing TRPC5-mCherry expression and PS externalization on the plasma membrane of HEK293 cell transfected with empty vector (**A**–**C**) or TRPC5-mCherry+PLSCR1 (**D**–**F**). The cells were treated with saline as a control (**A**, **D**), a hypotonic solution (**B**, **E**) or LaCl_3_ (100 μmol/L; **C** and **F**). (**G**–**H**) Summary data showing the PS externalized cells in percentage of total cells (FITC-positive). **G**: data from **A**–**C**; **H**: data from FITC channel in **D**–**F**. PS externalization was detected as green fluorescence via the annexin V-FITC assay. TRPC5 is detected as red fluorescence. Values are shown as the mean ± SEM (n = 3); *p < 0.05 for Control vs. Hypotonic or LaCl_3_ with a two-tailed unpaired Student’s *t* test.

Our previous study also showed that a hypotonic solution, LaCl_3_ or daidzein can activate TRPC5 [25]. When an empty vector (control) was transfected into HEK293 cells, activation of TRPC5 either with a hypotonic solution or with LaCl_3_ (100 μmol/L) only caused very weak/minimal PS externalization (Figure 2A–C, G). By contrast, in HEK293 cells co-transfected with TRPC5-mCherry and PLSCR1, activation of TRPC5 by a hypotonic solution or LaCl_3_ induced a very strong PS externalization (Figure 2D–F, H), indicating that overexpression of TRPC5 plus PLSCR1 substantially stimulated the PS externalization.

**Figure 3:**
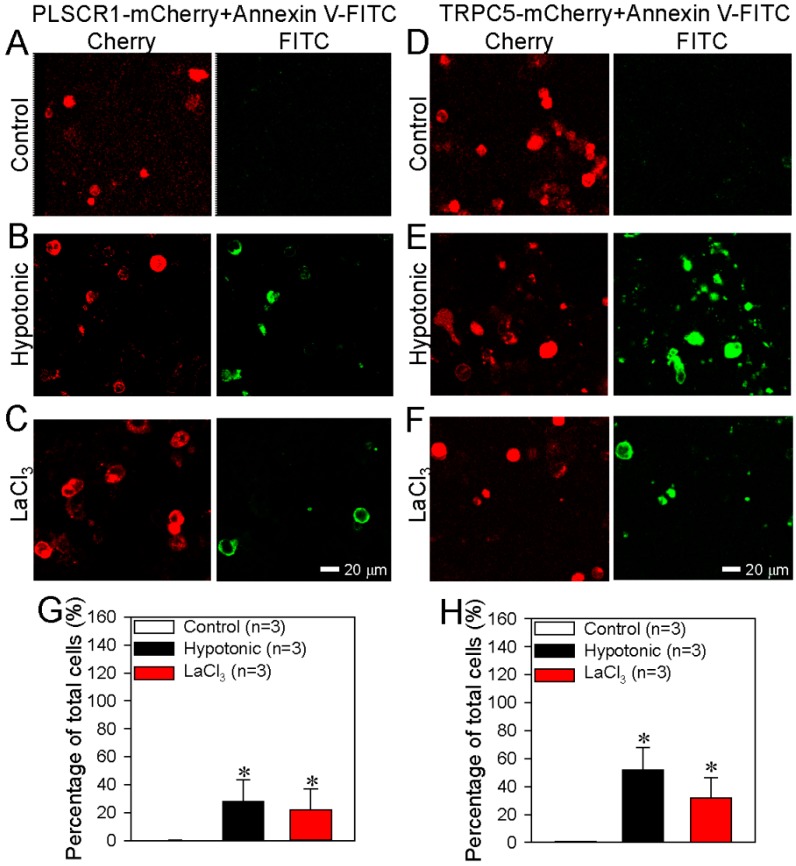
Effect of PLSCR1 alone or TRPC5 alone on phosphatidylserine (PS) externalization in HEK293 cells. (**A**–**F**) Representative images showing the expression of PLSCR1-mCherry or TRPC5-mCherry and PS externalization on the plasma membrane of HEK293 cells transfected with PLSCR1-mCherry alone (**A**–**C**) or TRPC5-mCherry alone (**D**–**F**). The cells were treated with saline (control) (**A**, **D**), hypotonic solution (**B**, **E**) or LaCl_3_ (100 μmol/L) (**C**, **F**). (**G**–**H**) Summary data showing the PS externalized cells (FITC-positive) in percentage of total cells (%). **G**: data from FITC channel in **A**–**C**; **H**: data from FITC channel in **D**–**F**. PS externalization was detected as green fluorescence via the annexin V-FITC assay. PLSCR1 (**A**–**C**) and TRPC5 (**D**–**F**) are detected as red fluorescence. Values are shown as the mean ± SEM (n = 3); *p < 0.05 for Control vs. Hypotonic or LaCl_3_ with a two-tailed unpaired Student’s *t* test.

**Figure 4:**
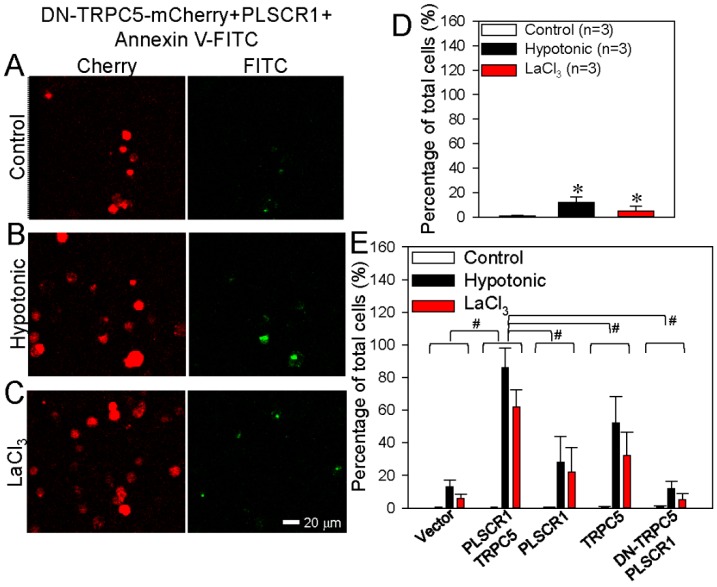
Effect of dominant negative TRPC5 (DN-TRPC5) on phosphatidylserine (PS) externalization in HEK293 cells. (**A**–**C**) Representative images showing DN-TRPC5-mCherry expression and PS externalization on the plasma membrane of HEK293 cell transfected with DN-TRPC5-mCherry+PLSCR1. The cells were treated with saline (control) (**A**), hypotonic solution (**B**), or LaCl_3_ (100 μmol/L) (**C**). (**D**) Summary data showing the PS externalized cells (FITC-positive) in percentage of total cells (%) in **A**–**C**. (**E**) Combined summary data showing the PS externalized cells (FITC-positive) in percentage of total cells (%) from Figure 2, Figure 3 and Figure 4. The data were from FITC channel. PS externalization was detected as green fluorescence via the annexin V-FITC assay. DN-TRPC5-mCherry was detected as red fluorescence. Values are shown as the mean ± SEM (n = 3); *p < 0.05 for Control vs. Hypotonic or LaCl_3_ with a two-tailed unpaired Student’s *t* test; ^#^*p* < 0.05, PLSCR1+TRPC5 vs. vector, PLSCR1, TRPC5 or DN-TRPC5+PLSCR1 with two-way ANOVA followed by the Tukey’s post-hoc comparison test.

In the cells transfected with PLSCR1-mCherry alone or with TRPC5-mCherry alone, activation of TRPC5 could still increase the PS externalization (Figure 3A–H), but the effect was much smaller than that in TRPC5-mCherry and PLSRC1 co-transfected cells (Figure 4E).

We also used DN-TRPC5, which contains mutation within the TRPC5 pore region thus abolishes the TRPC5 channel activity. Intriguingly, in HEK293 cells co-transfected with PLSCR1 and a dominant-negative TRPC5 construct (DN-TRPC5-mCherry), TRPC5 activation could hardly induce any PS externalization (Figure 4A–E), suggesting a critical importance of TRPC5 channel activity in promoting PS externalization. Taken together, these results strongly suggest that TRPC5 and PLSCR1 both play a role in promoting PS externalization. Note that native (un-transfected) HEK293 cells may contain low levels of endogenous TRPCs [47] and PLSCR1. This can explain why overexpression of PLSCR1 alone or TRPC5 alone could also induce moderate increase in PS externalization.

### 3.3. Physical Association of TRPC5 and PLSCR1 in Native Neurons

**Figure 5:**
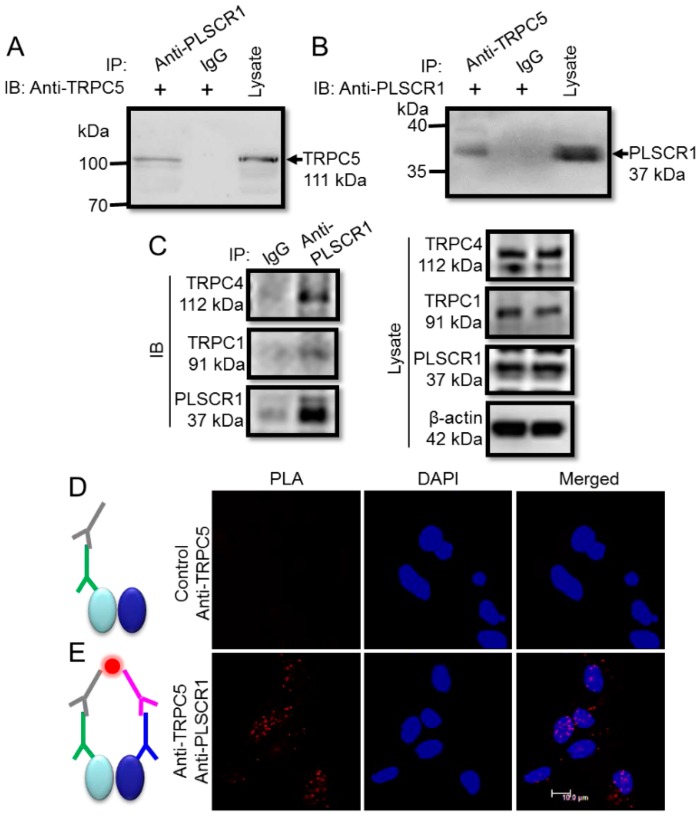
Association between TRPC5 and PLSCR1 in freshly isolated mouse cortical neurons. (**A**, **B**) Representative images of co-immunoprecipitation experiments. (**A**): IP, anti-PLSCR1 antibody or IgG; IB, anti-TRPC5 antibody. **B**, IP, anti-TRPC5 antibody or IgG; IB, anti-PLSCR1 antibody. Immunoblots of cell lysates were also shown on the right. (**A**), immunoblot with rabbit anti-TRPC5 antibody; (**B**), immunoblot with goat anti-PLSCR1 antibody). The experiments were repeated for three times. (**C**) Representative images of co-immunoprecipitation experiments. Right: IP, anti-PLSCR1 antibody or IgG; IB, anti-TRPC1, anti-TRPC4, or anti-PLSCR1 antibody. Left, Immunoblots of cell lysates were also shown. (**D**, **E**) In situ proximity ligation assay (PLA) to identify the association between TRPC5 and PLSCR1 in native neurons. Representative images were in the presence of both anti-TRPC5 and anti-PLSCR1 antibodies (**E**), or in the presence of anti-TRPC5 antibody alone (Control) (**D**). In **D** and **E**, gray sign: anti-goat PLA probe Plus, green sign: goat anti-TRPC5 antibody, pink sign: anti-rabbit PLA probe Minus, blue sign: rabbit anti-PLSCR1 antibody, red dot: PLA signal, left oval: TRPC5 protein, right oval: PLSCR1 protein. Nuclei were stained blue by DAPI.

Because TRPC5 is richly expressed in the nervous system, we next determined the functional role of the TRPC5-PLSCR1 complex in neurons. Co-immunoprecipitation assays was used to determine the physical association of TRPC5 and PLSCR1 in freshly isolated mouse cortical neurons. The immunoblotting results showed strong expression of TRPC5 and PLSCR1 in mouse cortical neurons (Figure 5A–B).

Moreover, the anti-PLSCR1 antibody could pull down TRPC5 from the lysates of mouse cortical neurons (Figure 5A) and vice versa (Figure 5B). The specificity of anti-TRPC5 antibody to TRPC5 has been reported previously by us [25]. As a negative control, preimmune IgG was also used in co-immunoprecipitation assays, and as expected, no co-immunoprecipitation products were detected (Figure 5A–B). In addition, the anti-PLSCR1 antibody could also pull down TRPC1 and TRPC4, suggesting the presence of heteromeric TRPC1-C4-C5 in neurons (Figure 5C).

To further confirm the physical interaction of TRPC5 and PLSCR1 in neurons, we used the method of in situ PLA, which can detect whether two proteins exist within a radius of <40 nm. The results showed many positive red fluorescence dots, indicative of TRPC5-PLSCR1 interaction sites, in mouse cortical neurons (Figure 5E). By contrast, in a negative control without anti-PLSCR1 antibody, no detectable red fluorescent dots were observed (Figure 5D). These results indicated that TRPC5 is physically associated with PLSCR1 as a complex in mouse cortical neurons. 

### 3.4. Activation of TRPC5 Stimulates Neuronal PS Externalization in Cerebral Slides

**Figure 6:**
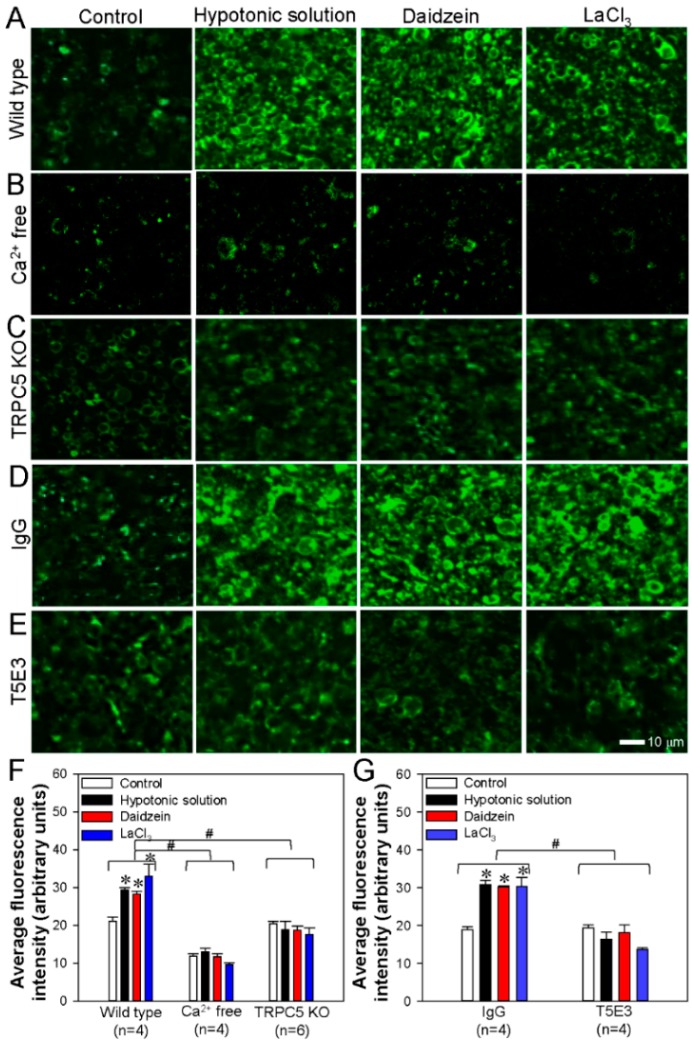
Activation of TRPC5 stimulates phosphatidylserine (PS) externalization in mouse cerebral neurons. (**A**–**E**) Representative images showing PS externalization (green fluorescence) as determined by an annexin V-FITC assay in neurons of cerebral slices obtained from wild-type (**A**) or TRPC5 knockout (TRPC5 KO) mice (**C**) or normal C57 mice (**B**, **D**, **E**). (**B**) in the absence of extracellular Ca^2+^ (Ca^2+^ free); (**D**) treated with IgG; (**E**) treated with a functional blocking antibody T5E3. (**F**, **G**) Summary of average fluorescence intensity (arbitrary units) of PS externalized neurons in cerebral slices obtained from wild-type (wild type, Ca^2+^ free) and TRPC5 KO mice (**F**) or normal C57 mice treated with IgG or T5E3 (**G**). In each group, cerebral slices were treated with hypotonic buffer, daidzein (100 μmol/L) or LaCl_3_ (100 μmol/L) for 10 min to activate TRPC5, or treated with saline as control. Values represent the mean ± SEM (n = 4-6); *p < 0.05, vs. control with a two-tailed unpaired Student’s *t* test; ^#^*p* < 0.05, wild type vs. Ca^2+^ free or TRPC5 KO, or IgG vs. T5E3 with two-way ANOVA followed by the Tukey’s post-hoc comparison test.

To examine the functional role of the TRPC5-PLSCR1 complex in PS externalization in native neurons, we used annexin V-FITC assays to visualize PS externalization in mouse cerebral slices. The mice were sacrificed with an overdose of CO_2_, and the cerebrum was immediately isolated and sliced. Beside hypotonic solution and LaCl_3_, daidzein (a genistein analog) can activate TRPC5 as well [48]. Our results showed that activation of TRPC5 by a hypotonic solution, daidzein, or LaCl_3_ strongly increased green fluorescence signals indicative of PS externalization in cerebral slices derived from wild-type mice (Figure 6A,F). In the absence of extracellular Ca^2+^, hypotonic solution, daidzein or LaCl_3_ failed to induce significant PS externalization (Figure 6B,F). In comparison, these treatments induced much weaker PS externalization in the brain slides derived from TRPC5 KO mice (Figure 6C,F).

To further confirm these findings, we used a functional blocking antibody T5E3 to block the Ca^2+^ permeation pore of TRPC5 [25]. The specificity of T5E3 to TRPC5 in neuronal tissues has been reported previously by us [35]. In the presence of T5E3, application of a hypotonic solution, daidzein, or LaCl_3_ could no longer induce PS externalization in the cerebral slices derived from wild-type mice (Figure 6E,G). As a control, when preimmune IgG was applied, the activation of TRPC5 resulted in substantial PS externalization (Figure 6D,G). Overall, these results indicated that the Ca^2+^ influx mediated by TRPC5 is critical for the PS externalization in mouse cerebral neurons.

### 3.5. Spatial Proximity and Association of TRPC5 and PLSCR1 on HEK293 Plasma Membrane

**Figure 7:**
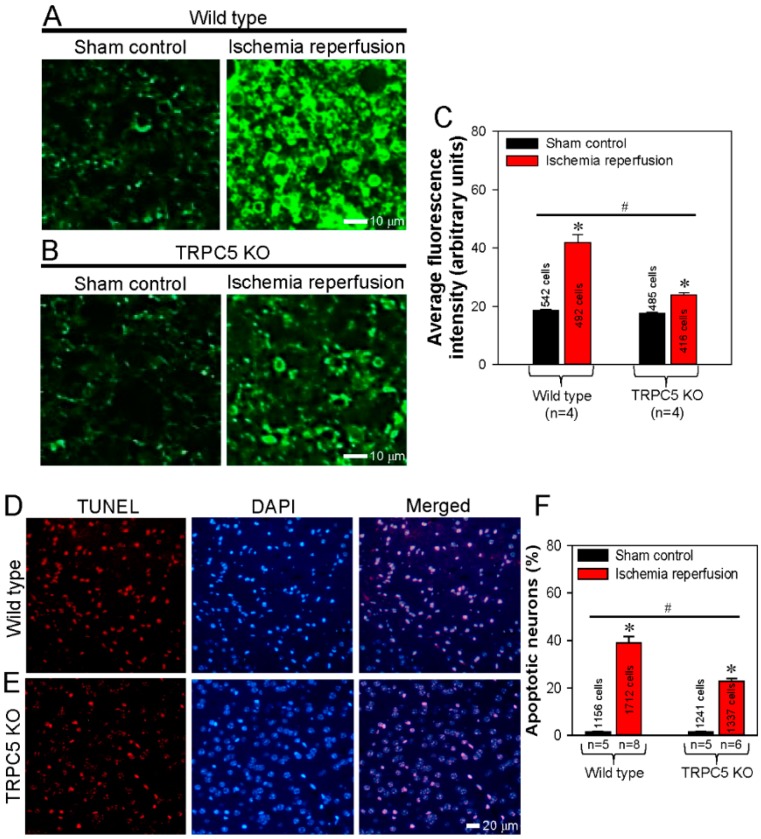
Knockout of TRPC5 protects cerebral neurons from ischemia-reperfusion-induced apoptosis. (**A**, **B**) Representative images and summarized data showing phosphatidylserine (PS) externalization as determined using an annexin V-FITC assay in neurons of cerebral slices obtained from wild-type (**A**, **C**) and TRPC5 knockout (TRPC5 KO) (**B**, **C**) mice subjected to sham surgery (sham control) or cerebral ischemia-reperfusion. (**C**) Summary of average fluorescence intensity (arbitrary units) of PS externalized neurons in cerebral slices obtained from wild-type and TRPC5 KO mice subjected to sham surgery (control) or cerebral ischemia-reperfusion. (**D**, **E**) Following surgery, TUNEL assays were performed to visualize apoptotic mouse cerebral neurons as red fluorescence signals in the neurons of cerebral slices obtained from wild-type (**D**) or TRPC5 KO (**E**) mice subjected to cerebral ischemia-reperfusion. Cell nuclei were stained blue with DAPI. Merged images show apoptotic signals overlapping with cell nuclei. (**F**) Summary of the percentages of apoptotic neurons in cerebral slices obtained from wild-type and TRPC5 KO mice subjected to sham surgery (control) or cerebral ischemia–reperfusion. Values represent the mean ± SEM (n = 5-8); *p < 0.05, control vs. ischemia-reperfusion with a two-tailed unpaired Student’s *t* test; ^#^*p* < 0.05, wild-type ischemia-reperfusion vs. TRPC5 KO ischemia–reperfusion with two-way ANOVA followed by the Tukey’s post-hoc comparison test.

Ischemia-reperfusion is a common cause of cephaledema and neuronal apoptosis in the central nervous system. The externalization of PS is indicative of early cell apoptosis. Hence, the high expression of TRPC5 in neurons and its close association with PLSCR1 inspired us to examine the functional role of TRPC5 in inducing neuronal apoptosis in mouse model of ischemia-reperfusion. Cerebral slides were prepared from wild-type mice that were subjected to ischemia-reperfusion or sham operation as control. PS externalization was visualized as green fluorescence signals using annexin V-FITC assay. The results showed that, in wild-type mice, ischemia-reperfusion induced strong PS externalization in cerebral slices compared to those of sham control (Figure 7A,C).

By contrast, the PS externalization induced by ischemia-reperfusion was much weaker in the cerebral slices derived from TRPC5 KO mice (Figure 7B,C). These results suggest that knockout of TRPC5 reduced the neuronal apoptosis induced by ischemia-reperfusion. To further verify this hypothesis, we performed TdT-mediated dUTP nick-end labeling (TUNEL) assays in which apoptotic cells were stained red, and cell nuclei were stained blue with DAPI. In cerebral slides prepared from ischemia-reperfusion mice, there were more TUNEL-positive apoptotic neurons in the samples from wild-type mice than in those from TRPC5 KO mice (Figure 7D–F). There were almost no TUNEL-positive apoptotic neurons in cerebral slides from sham-operated control mice (Figure 7F). These results demonstrated that TRPC5 knockout protected cerebral neurons from apoptosis induced ischemia-reperfusion.

## 4. Discussion

The two main findings of the present study are that TRPC5 physically associates with PLSCR1 to facilitate PS externalization in HEK293 cells and mouse cerebral neurons, and that inhibiting TRPC5 may have a protective effect in cerebral ischemia-reperfusion injury. The evidence for these findings is as follows: (1) FRET assays and co-immunoprecipitation experiments demonstrated a physical association of TRPC5 with PLSCR1 in HEK293 cells. (2) Following the transfection of both TRPC5 and PLSCR1, activation of TRPC5 greatly increased PS externalization in HEK293 cells. In contrast, TRPC5 activation had much smaller effect on the PS externalization in HEK293 cells that were transfected with TRPC5 alone or PLSCR1 alone. In HEK293 cells that were transfected with dominant negative TRPC5 construct, TRPC5 activation only had very smaller/minimal effect on the PS externalization. (3) Co-immunoprecipitation assays and in situ PLA demonstrated the physical association of TRPC5 with PLSCR1 in mouse cerebral neurons. Activation of TRPC5 induced marked PS externalization in cerebral neurons of wild-type mice but not in the neurons from TRPC5 KO mice. In the absence of extracellular Ca^2+^ or in the presence of the TRPC5 blocking antibody T5E3, activation of TRPC5 failed to induce PS externalization in cerebral neurons of wild-type mice. (4) Following cerebral ischemia–reperfusion, strong PS externalization and neuronal apoptosis were recorded in the brain slides of wild-type mice not in those of TRPC5 KO mice. 

In the lipid bilayer of the cell membrane, PS is normally asymmetrically distributed in the cytosolic leaflet [49], where PS facilitates the interaction of membrane proteins on the cytosolic surface, such as protein kinase C and Na^+^/K^+^-ATPase [49]. PS also participates in membrane fusion events, such as exocytosis, in a Ca^2+^-dependent manner. This asymmetrical distribution of PS on the lipid bilayer is primarily caused by activity of unidirectional lipid transporter, especially flippase and floppase [49], and is important for normal cell function and survival. However, when cells are activated by a localized Ca^2+^ concentration rise, scramblases can move PS to the outer leaflet within minutes [50], causing the collapse of PS membrane asymmetry. PS exposure to the outer leaflet may attract macrophages, resultant in macrophage phagocytosis of the PS exposure abnormal cells [51,52,53,54]. Furthermore, the exposure of PS in blood platelets may facilitate the interaction of platelets with coagulating proteins during blood coagulation [55,56,57]. In the present study, we investigated the physical interaction of TRPC5 with PLSCR1 and explored the role of TRPC5 in PS externalization. By using FRET assay, we found close proximity between the carboxyl terminal of TRPC5 and the intracellular carboxyl terminal of PLSCR1. In functional studies, co-transfection of TRPC5 with PLSCR1 caused very strong PS externalization in HEK293 cells, whereas co-transfection of dominant negative TRPC5 with PLSCR1 only had minimal effect on PS externalization. A likely scenario is that TRPC5, through its localized Ca^2+^ influx, effectively activates PLSCR1 to translocate PS from the cytosolic leaflet to the outer leaflet.

Note that although we have demonstrated the physical association of TRPC5 and PLSCR1, there is still not sufficient evidence for the absolute requirement of such physical association in the PS externalization. However, it is well documented that Ca^2+^ signaling components often have physical coupling to facilitate the interaction among different components for eventually cellular responses [58,59]. It is conceivable that the physical association of TRPC5 with PLSCR1 may facilitate their function in the PS externalization.

TRPC5 is abundantly expressed in native neurons [60]. Thus, we further investigated the role of the TRPC5-PLSCR1 complex in PS externalization in the cerebral neurons of mice. Our results demonstrated a similar physical interaction of TRPC5 with PLSCR1 in mouse cerebral neurons. Furthermore, activation of TRPC5 by hypotonicity or La^3+^ induced strong PS externalization in these neurons. Previous studies have shown that cerebral ischemia and ischemia–reperfusion can cause severe neuronal damage, including cephaledema and neuronal apoptosis [61]. To investigate the function of TRPC5 in ischemia–reperfusion injury, we conducted experiments using TRPC5 KO mice. Note that TRPC5 KO mice show no abnormalities in weight, neurological reflexes, sensorimotor responses, or basic motor functions but appear less anxious in response to innately aversive stimuli [32]. In our results, compared with wild-type mice, TRPC5 KO mice showed substantially less PS externalization and less apoptosis of cerebral neurons, suggesting that TRPC5 KO mice experienced less injury following ischemia-reperfusion and might have a better prognosis.

## 5. Conclusions

We demonstrated that TRPC5 physically interacts with PLSCR1 and that activation of TRPC5 induces PS externalization in TRPC5-PLSCR1 overexpressing HEK293 cells and native mouse cerebral neurons. However, when the TRPC5 pore region was blocked by a specific antibody in wild-type mice or when TRPC5 KO mice were used, activation of TRPC5 did not caused noticeable PS externalization. Additionally, using a mouse model of ischemia-reperfusion injury, we found that TRPC5 KO mice had fewer neurons undergoing PS exposure and apoptosis, suggesting a protective effect of TRPC5 inhibition. Therefore, we propose that TRPC5 physically interacts with PLSCR1 as a signaling complex to mediate PS externalization and apoptosis in neurons and that TRPC5 may play a role in cerebral ischemia-reperfusion injury.
